# Supplementary figures and images for: Resurrection of the ancestral RH5 invasion ligand provides a molecular explanation for the origin of P. falciparum malaria in humans
Source: PLoS Biol. 2019 Oct 15;17(10):e3000490. doi: 10.1371/journal.pbio.3000490 (PMC6793842; doi:10.1371/journal.pbio.3000490)

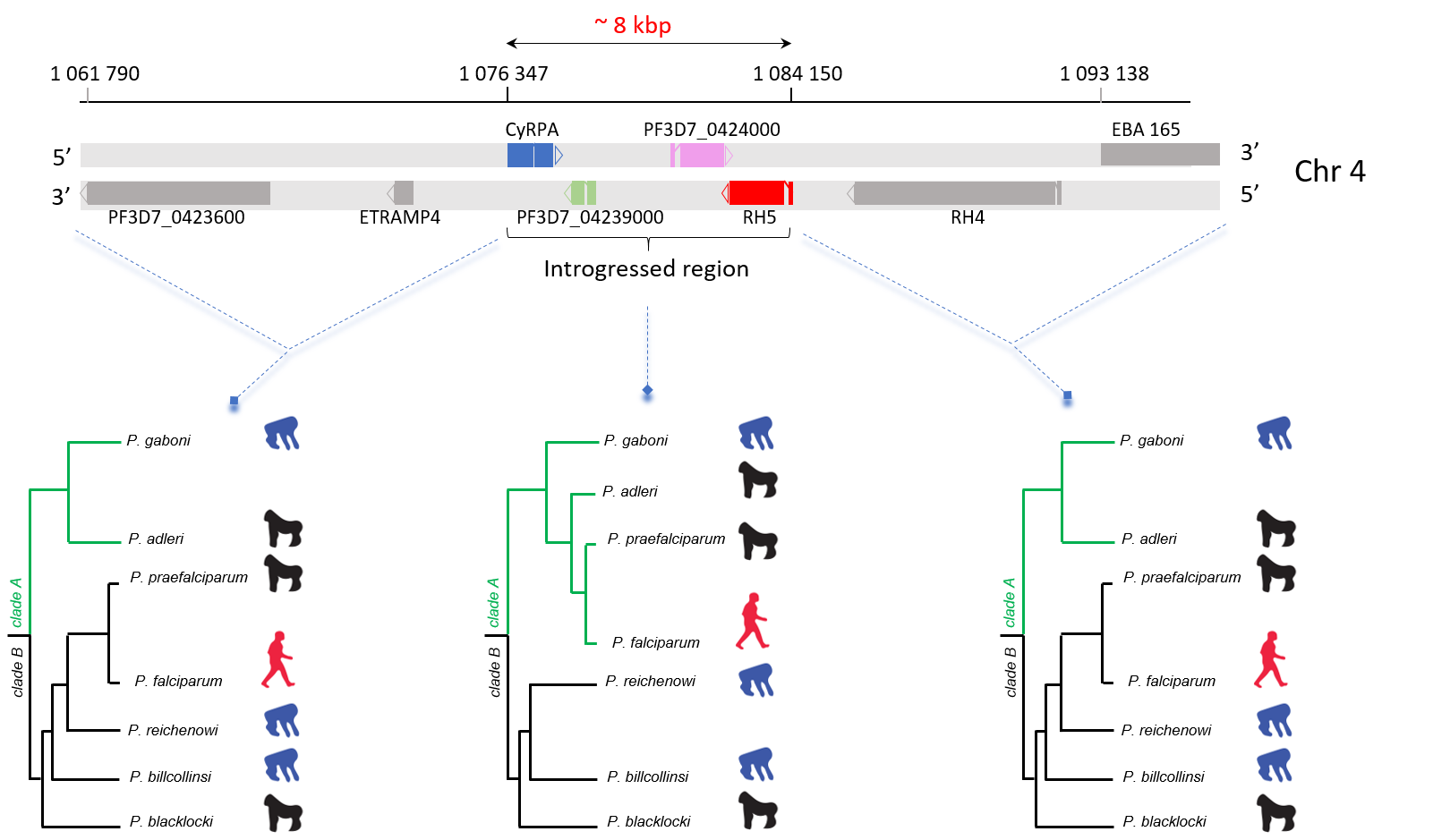

Supplement: S1 Fig — Each DNA strand is represented by gray bars, and open reading frames encoding the named protein products are colored. The scale indicates the equivalent position in the P. falciparum 3D7 reference genome. The phylogenetic topologies calculated for the introgressed and flanking sequences are provided, illustrating the extent and origin of the introgressed region. (TIF) [file pbio.3000490.s001.tif]

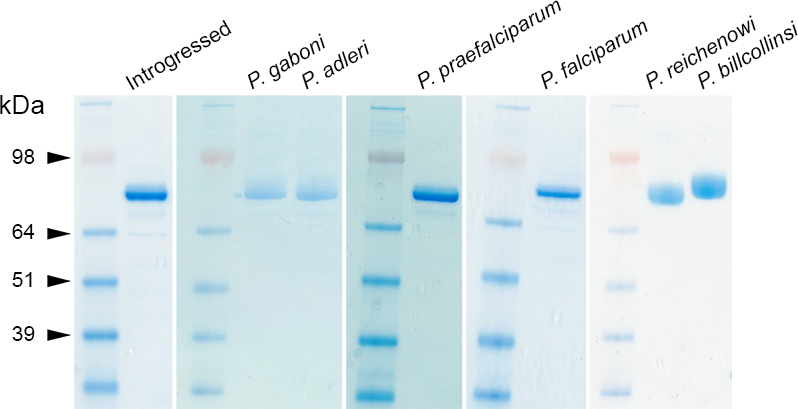

Supplement: S2 Fig — The indicated Laverania RH5 proteins were expressed in HEK293 cells as secreted recombinant proteins with a Cd4(d3+4)-His6+ tag and purified by immobilized Ni2+ ion chromatography. Proteins were resolved by SDS-PAGE under reducing conditions and stained with Coomassie brilliant blue. Expected molecular masses: introgressed RH5, 84.9 kDa; P. gaboni, 85.2 kDa; P. adleri, 84.9 kDa; P. praefalciparum, 85.7 kDa; P. falciparum, 84.7 kDa; P. reichenowi, 82.5 kDa; P. billcollinsi, 84.9 kDa. Original unprocessed gels can be found in S1 Data. Cd4(d3+4)-His6+, Ig-like domains 3 and 4 of rat CD4; RH5, reticulocyte-binding protein homologue 5. (TIF) [file pbio.3000490.s002.tif]

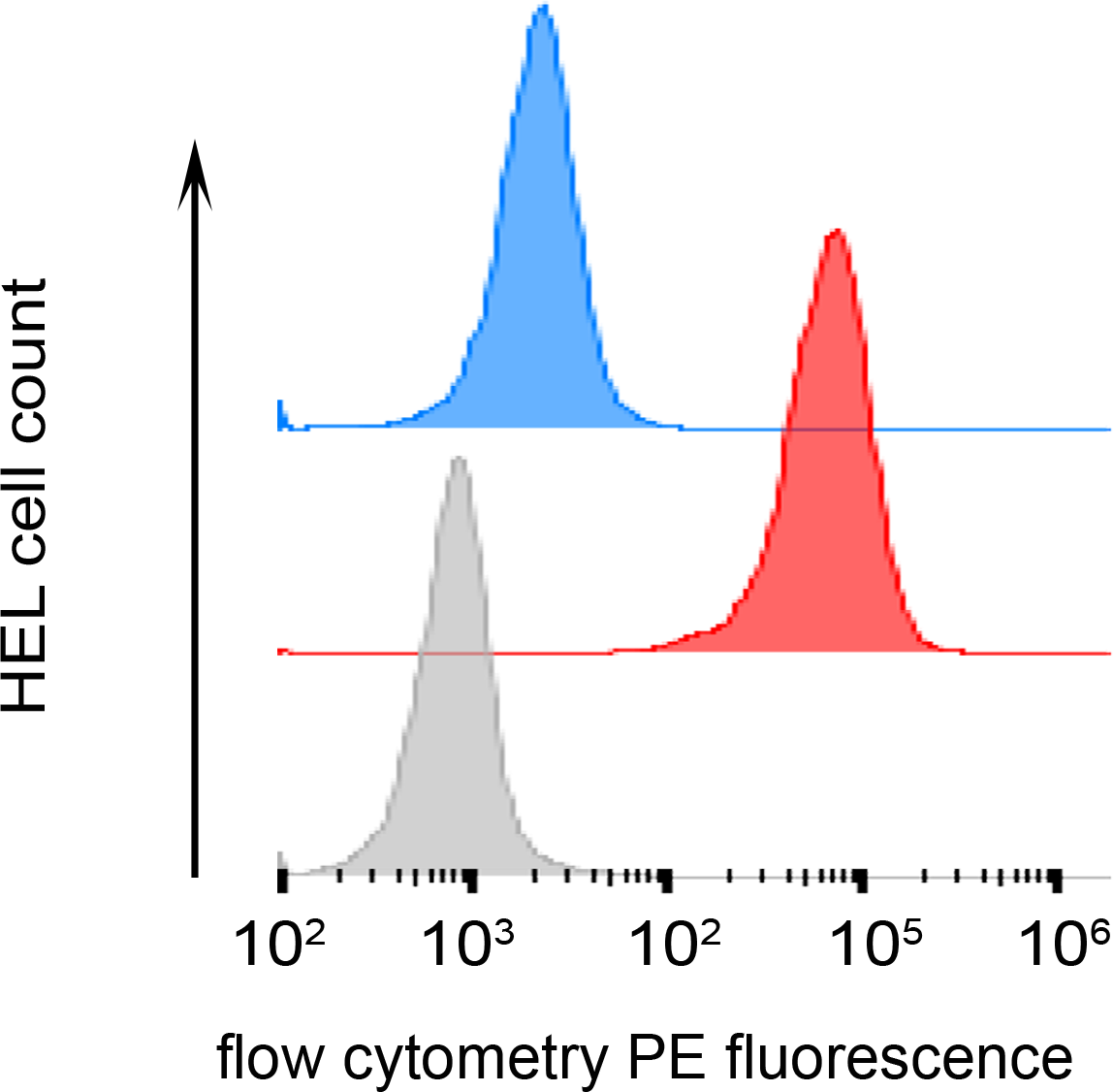

Supplement: S3 Fig — Binding specificities of Laverania parasite RH5 proteins with human basigin were confirmed by cell binding experiments. The introgressed RH5 protein was expressed as an enzymatically monobiotinylated protein, purified, and clustered around a streptavidin-PE conjugate to create an avid RH5 labeled binding probe before presenting to basigin-expressing HEL cells. Specificity was demonstrated by showing that RH5 probe binding activity was abolished by preincubating the cells with an anti-basigin mAb that blocks RH5 binding (Ab1—blue histograms) compared with a cell-binding isotype-matched anti-CD59 mAb (red). Control is streptavidin-PE alone (gray). Summary numerical data are provided in S1 Data; gating strategy and original .fcs files in S2 Data. Ab1, anti-basigin mAb; HEL, human erythroid-like; mAb, monoclonal antibody; PE, phycoerythrin; RH5, reticulocyte-binding protein homologue 5. (TIF) [file pbio.3000490.s003.tif]

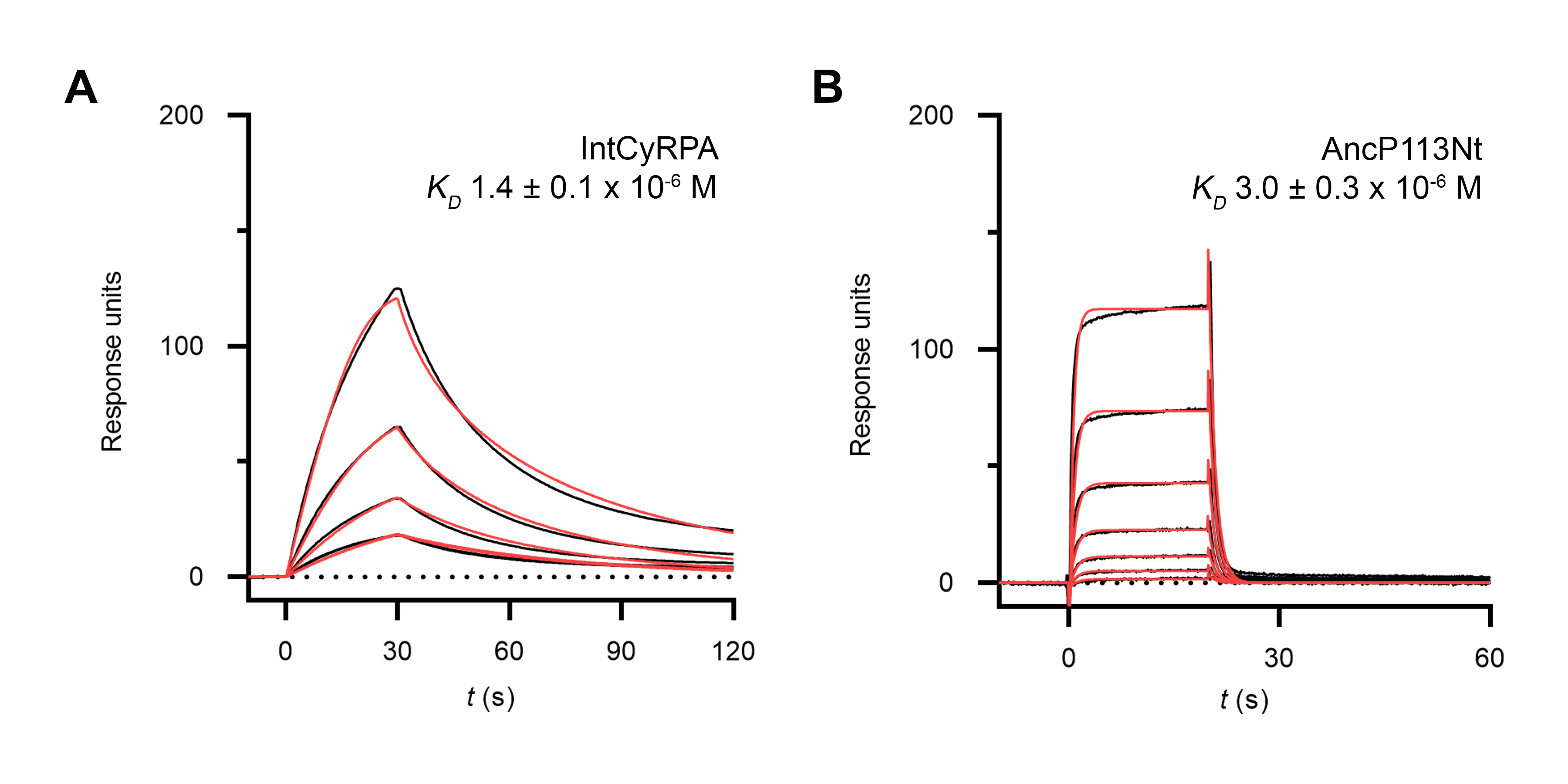

Supplement: S4 Fig — SPR traces showing that the introgressed RH5 protein is able to directly interact with the introgressed CyRPA (A) and with the known RH5 binding site in the N terminus of P113 (B). Both the introgressed CyRPA and N terminus of the ancestral P113 were expressed as soluble enzymatically monobiotinylated proteins and 800 RU and 600 RU were captured, respectively, on the surface of a streptavidin-coated sensor chip. Serial dilutions of purified introgressed RH5 were injected at 100 μL/minute over IntCyRPA (full-length introgressed RH5) and P113Nt (N terminus of introgressed RH5), respectively, and the biophysical binding parameters of the interaction calculated by fitting the binding data (black) to a simple 1:1 binding model (red). Underlying numerical data can be found in S1 Data. CyRPA, cysteine-rich protective antigen; IntCyRPA, introgressed ancestral CyRPA; P113Nt, P113 N-terminal domain; RH5, reticulocyte-binding protein homologue 5; SPR, surface plasmon resonance. (TIF) [file pbio.3000490.s004.tif]

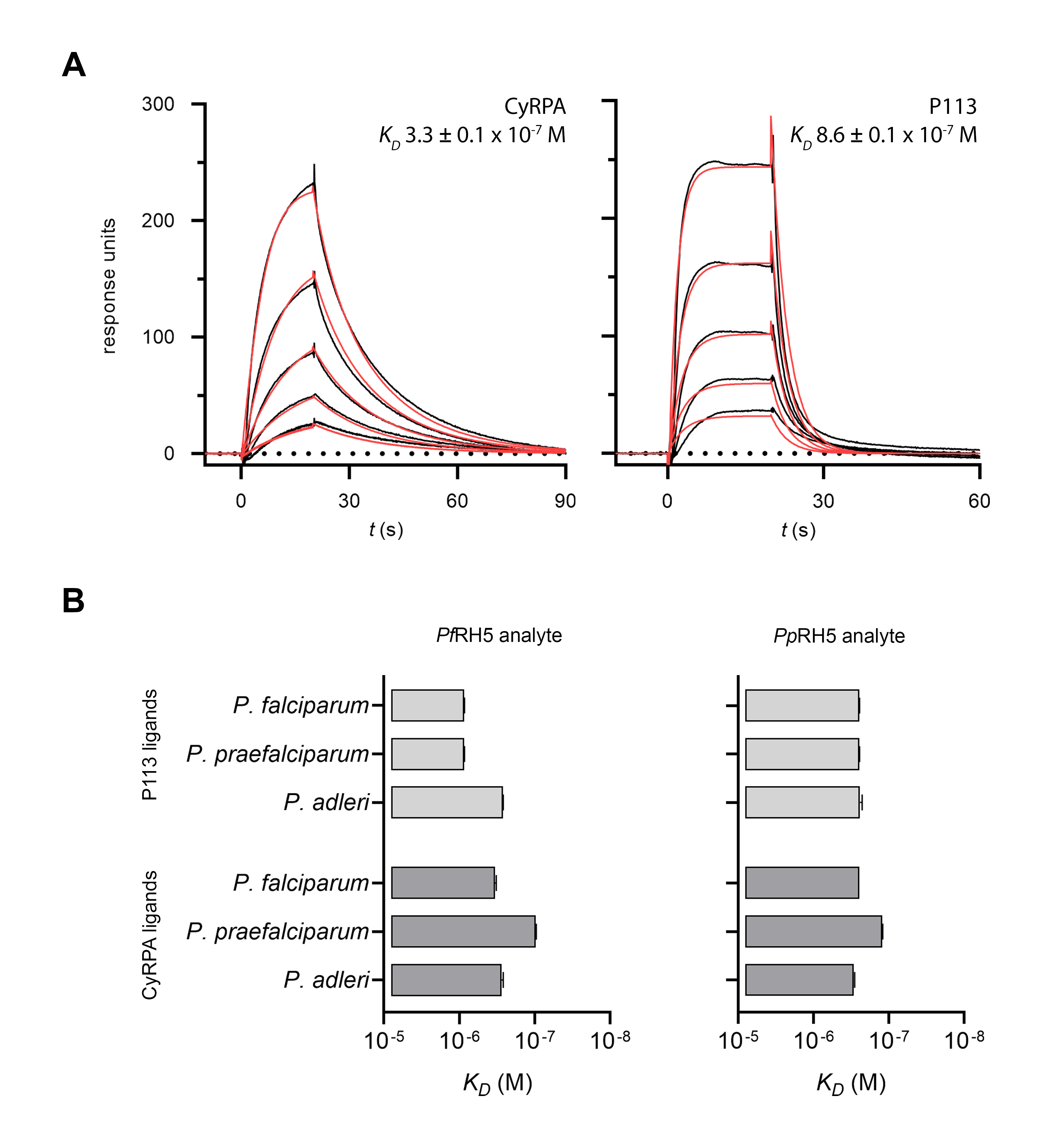

Supplement: S5 Fig — (A) Representative SPR sensorgrams quantifying the RH5-CyRPA (left panel) and RH5-P113 (right panel) interactions used to calculate the summary data shown in (B). In this example, serial dilutions of P. falciparum RH5 were used as the analyte with enzymatically monobiotinylated P. falciparum CyRPA and P113 immobilized on a streptavidin-coated sensor chip. Biophysical binding parameters were calculated by fitting the raw binding data (black) to a simple 1:1 binding model (red). (B) A summary of affinity measurements between P. falciparum (Pf) and P. praefalciparum (Pp) RH5 and P. falciparum, P. praefalciparum, and P. adleri CyRPA and P113. The equilibrium dissociation constants (KD) for each interaction was calculated from 1:1 fits to the SPR binding data and plotted. Bars represent means ± s.e.m. from at least five different analyte concentrations. Underlying numerical data can be found in S1 Data. CyRPA, cysteine-rich protective antigen; Pf, P. falciparum; Pp, P. praefalciparum; RH5, reticulocyte-binding protein homologue 5; SPR, surface plasmon resonance. (TIF) [file pbio.3000490.s005.tif]

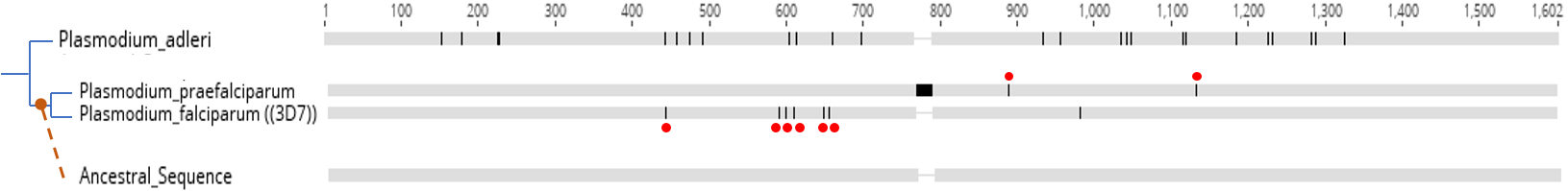

Supplement: S6 Fig — Black bars indicate a difference with the ancestral sequence. The red dots indicate a nonsynonymous substitution in P. falciparum and P. praefalciparum. RH5, reticulocyte-binding protein homologue 5. (TIF) [file pbio.3000490.s006.tif]

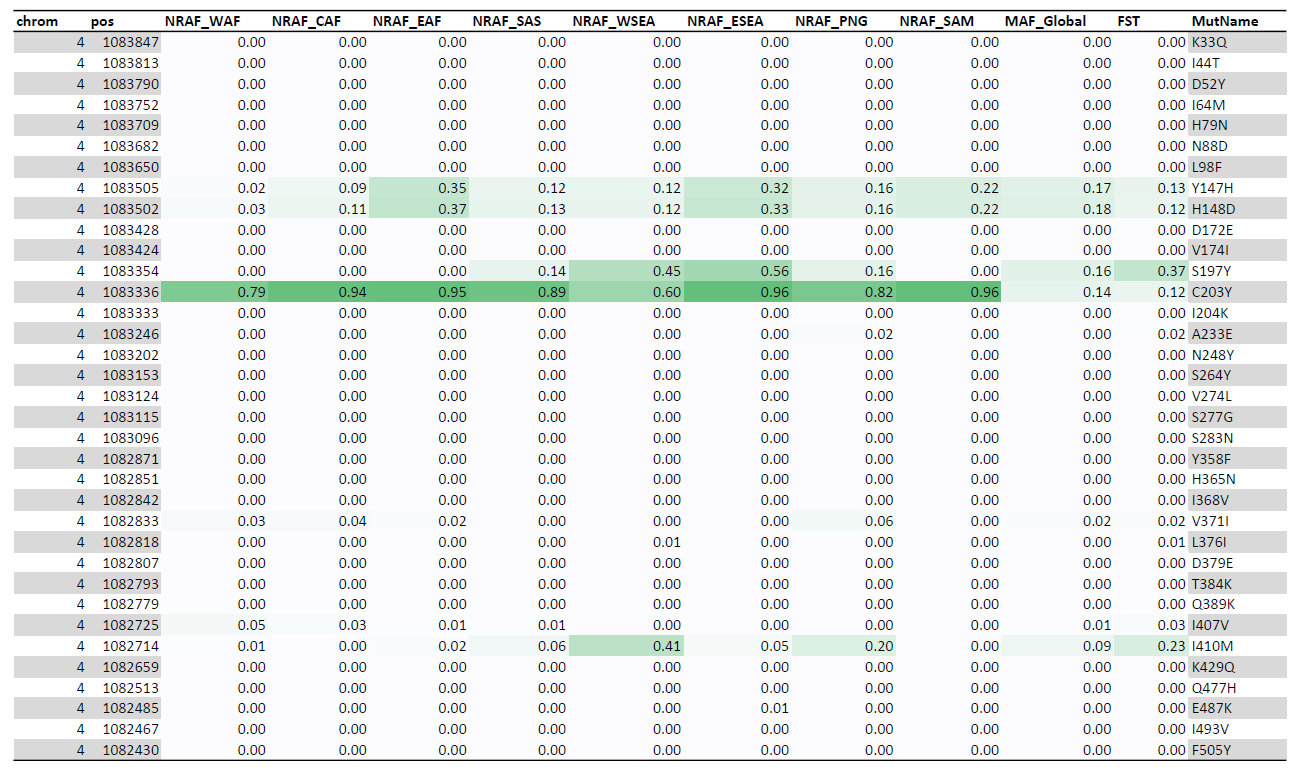

Supplement: S2 Table — The introgressed H148 allele is present in 18% of P. falciparum isolates, while the Y197 allele dominates in Southeast Asia. The Y203 allele is dominant globally (86% of sequenced isolates), making the 3D7 strain unrepresentative for this position. The H200, R216, and Q219 present in the calculated introgressed RH5 sequence have not been detected in extant sequenced P. falciparum populations. CAF, Central Africa; EAF, East Africa; ESEA, East Southeast Asia; FST, population differentiation statistic; MAF, global allele frequency; NRAF, non-reference allele frequencies; PNG, Papua New Guinea; SAM, South America; SAS, South Asia; WAF, West Africa; WSEA, West South East Asia; RH5, reticulocyte-binding protein homologue 5. (TIF) [file pbio.3000490.s008.tif]

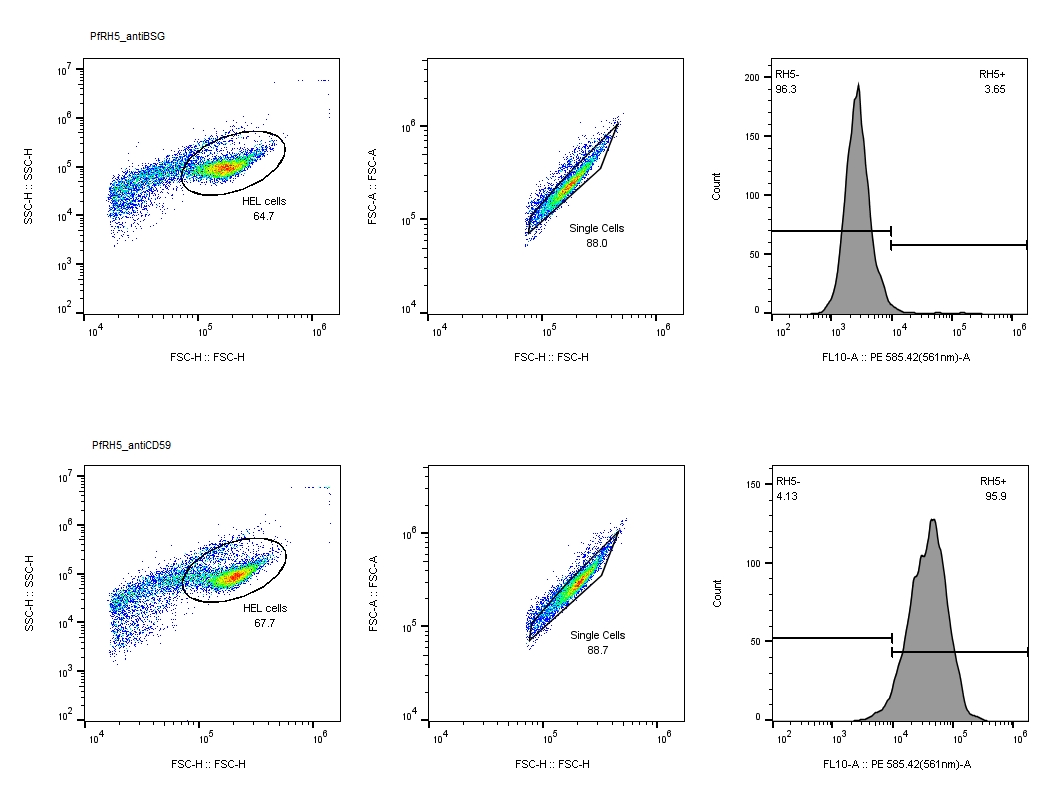

Supplement: S2 Data — (ZIP) [file pbio.3000490.s010.zip › FACS_Data/Fig2B_S3_Gating_strategy.jpg]
