# Supplementary material for: Resurrection of the ancestral RH5 invasion ligand provides a molecular explanation for the origin of P. falciparum malaria in humans
Source: PLoS Biol. 2019 Oct 15;17(10):e3000490. doi: 10.1371/journal.pbio.3000490 (PMC6793842; doi:10.1371/journal.pbio.3000490)
Supplement: Attachment — Submitted filename: Galaway_PbP_PLoSB.pdf [file pbio.3000490.s011.pdf]

We thank the reviewers for their valuable time in reviewing our manuscript entitled "*Resurrection of the ancestral RH5 invasion ligand provides a molecular explanation for the origin of* P. falciparum *malaria in humans*". We were pleased with the overall positive response to our manuscript. We have provided a point-by-point response to the referees' comments below and provided details on how we have revised the manuscript accordingly.

Reviewer #1:

Galaway and co-authors describe their efforts to recreate and phenotype an ancestral version of a protein (Rh5) that mediates red blood cell invasion by malaria parasites and is thought to have been a crucial player in the origin of P. falciparum as a human parasite through an introgression event from a gorilla parasite lineage. This approach taken by the authors to express extant and ancestral versions of the Rh5 protein is elegant, and the authors employ both SPR and cell-based assays to produce robust phenotype profiles. This work is an important addition to the exciting recent advances in understanding the origins of human malaria and the role of red blood cell invasion in host tropism.

The manuscript is clearly written and the authors' conclusions are generally well supported. I have the following questions and suggestions for minor clarifications:

1) The ancestral gene reconstruction for IntRH5 is included in a supplemental table, but a cursory description of how divergent it is from extant Pfal/Padleri/Ppraefalciparum sequences in the main text would be useful. How many amino acid subs from extant species? It could be useful, for example, to see a small tree showing the subs that have subsequently occurred on the Pfal/Pprae lineages since introgression to understand the rough magnitude of divergence that has subsequently occurred.

As suggested by the reviewer, we have prepared a figure showing the divergence between the ancestral introgressed sequence and the extant sequences of *P. adleri*, *P. praefalciparum* and *P. falciparum* of RH5. This figure is shown below and has been added to the revised version of the manuscript as Fig. S6.

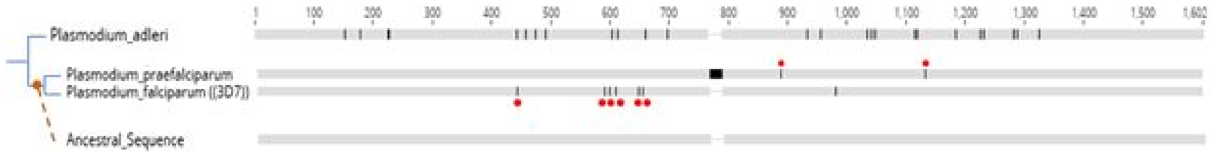

Figure S6. Alignment of the RH5 sequence obtained from the extant species and the calculated ancestral sequence. Black bars indicate a difference with the ancestral sequence. The red dots indicate a non-synonymous substitution in *P. falciparum* and *P. praefalciparum*.

2) On a similar note, near the end of Results the authors mention 6 AA differences between the introgressed and reference 3D7 assembly sequence for Rh5. It would be helpful to clarify here whether 3D7 is expected to reflect ancestral or derived alleles at these positions for P. falciparum, given that Rh5 is polymorphic in contemporary parasite populations (albeit lowly relative to other invasion genes).

To answer this question, we have downloaded the latest RH5 polymorphism data from MalariaGEN which is shown in the table below. These data show that of the six amino acid differences between the calculated ancestral RH5 and the reference 3D7 RH5, the H148 difference is present in 18% of *P. falciparum* isolates while the Y197 polymorphism dominates in South East Asia (which was already discussed in the text). The Y203 allele is dominant globally (present in 86% of sequenced isolates) making the 3D7 strain - which encodes a cysteine at this position - unrepresentative. Conversely, the residues H200, R216 and Q219 present in the calculated introgressed RH5 sequence have not been detected in extant sequenced *P. falciparum* populations. We agree with the reviewer that these are important data for comparison and so we have included the table in our revised manuscript as Table S2 (shown below).

chrom	pos	NRAF_WAF	NRAF_CAF	NRAF_EAF	NRAF_SAS	NRAF_WSEA	NRAF_ESEA	NRAF_PNG	NRAF_SAM	MAF_Global	FST	MutName
4	1083847	0.00	0.00	0.00	0.00	0.00	0.00	0.00	0.00	0.00	0.00	K33Q
4	1083813	0.00	0.00	0.00	0.00	0.00	0.00	0.00	0.00	0.00	0.00	I44T
4	1083790	0.00	0.00	0.00	0.00	0.00	0.00	0.00	0.00	0.00	0.00	D52Y
4	1083752	0.00	0.00	0.00	0.00	0.00	0.00	0.00	0.00	0.00	0.00	I64M
4	1083709	0.00	0.00	0.00	0.00	0.00	0.00	0.00	0.00	0.00	0.00	H79N
4	1083682	0.00	0.00	0.00	0.00	0.00	0.00	0.00	0.00	0.00	0.00	N88D
4	1083650	0.00	0.00	0.00	0.00	0.00	0.00	0.00	0.00	0.00	0.00	L98F
4	1083505	0.02	0.09	0.35	0.12	0.12	0.32	0.16	0.22	0.17	0.13	Y147H
4	1083502	0.03	0.11	0.37	0.13	0.12	0.33	0.16	0.22	0.18	0.12	H148D
4	1083428	0.00	0.00	0.00	0.00	0.00	0.00	0.00	0.00	0.00	0.00	D172E
4	1083424	0.00	0.00	0.00	0.00	0.00	0.00	0.00	0.00	0.00	0.00	V174I
4	1083354	0.00	0.00	0.00	0.14	0.45	0.56	0.16	0.00	0.16	0.37	S197Y
4	1083336	0.79	0.94	0.95	0.89	0.60	0.96	0.82	0.96	0.14	0.12	C203Y
4	1083333	0.00	0.00	0.00	0.00	0.00	0.00	0.00	0.00	0.00	0.00	I204K
4	1083246	0.00	0.00	0.00	0.00	0.00	0.00	0.02	0.00	0.00	0.02	A233E
4	1083202	0.00	0.00	0.00	0.00	0.00	0.00	0.00	0.00	0.00	0.00	N248Y
4	1083153	0.00	0.00	0.00	0.00	0.00	0.00	0.00	0.00	0.00	0.00	S264Y
4	1083124	0.00	0.00	0.00	0.00	0.00	0.00	0.00	0.00	0.00	0.00	V274L
4	1083115	0.00	0.00	0.00	0.00	0.00	0.00	0.00	0.00	0.00	0.00	S277G
4	1083096	0.00	0.00	0.00	0.00	0.00	0.00	0.00	0.00	0.00	0.00	S283N
4	1082871	0.00	0.00	0.00	0.00	0.00	0.00	0.00	0.00	0.00	0.00	Y358F
4	1082851	0.00	0.00	0.00	0.00	0.00	0.00	0.00	0.00	0.00	0.00	H365N
4	1082842	0.00	0.00	0.00	0.00	0.00	0.00	0.00	0.00	0.00	0.00	I368V
4	1082833	0.03	0.04	0.02	0.00	0.00	0.00	0.06	0.00	0.02	0.02	V371I
4	1082818	0.00	0.00	0.00	0.00	0.01	0.00	0.00	0.00	0.00	0.01	L376I
4	1082807	0.00	0.00	0.00	0.00	0.00	0.00	0.00	0.00	0.00	0.00	D379E
4	1082793	0.00	0.00	0.00	0.00	0.00	0.00	0.00	0.00	0.00	0.00	T384K
4	1082779	0.00	0.00	0.00	0.00	0.00	0.00	0.00	0.00	0.00	0.00	Q389K
4	1082725	0.05	0.03	0.01	0.01	0.00	0.00	0.00	0.00	0.01	0.03	I407V
4	1082714	0.01	0.00	0.02	0.06	0.41	0.05	0.20	0.00	0.09	0.23	I410M
4	1082659	0.00	0.00	0.00	0.00	0.00	0.00	0.00	0.00	0.00	0.00	K429Q
4	1082513	0.00	0.00	0.00	0.00	0.00	0.00	0.00	0.00	0.00	0.00	Q477H
4	1082485	0.00	0.00	0.00	0.00	0.00	0.01	0.00	0.00	0.00	0.00	E487K
4	1082467	0.00	0.00	0.00	0.00	0.00	0.00	0.00	0.00	0.00	0.00	I493V
4	1082430	0.00	0.00	0.00	0.00	0.00	0.00	0.00	0.00	0.00	0.00	F505Y

Table S2. Publically available MalariaGEN data showing non-reference allele frequencies (NRAF) in the P. falciparum population for RH5. WAF = West Africa; CAF = Central Africa; EAF = East Africa; SAS = South Asia; WSEA = West South East Asia; ESEA

= East South East Asia; PNG = Papua New Guinea; SAM = South America; MAF = global allele frequency; FST = population differentiation statistic. The introgressed H148 allele is present in 18% of *P. falciparum* isolates while the Y197 allele dominates in South East Asia. The Y203 allele is dominant globally (86% of sequenced isolates) making the 3D7 strain unrepresentative for this position. The H200, R216 and Q219 present in the calculated introgressed RH5 sequence have not been detected in extant sequenced *P. falciparum* populations.

3) The Discussion does not remark upon an observation one can make by comparing Figures 2A and 4B: that contemporary Pprae Rh5 is better at binding both human and gorilla basigen than the inferred introgressed allele (IntRH5). IntRH5 appears to have been a fairly poor binder to gorilla basigen, in fact, suggesting that subsequent adaption within the Ppra lineage may have been necessary to achieve more effective infection of gorillas following an introgression event and selective sweep driven by the capacity to successfully infect humans. This is a surprising finding, perhaps indicating that the introgression event subsumed a presumed pre-existing chimp host tropism in the Pf/Pprae ancestral lineage due to the evolutionary advantage arising from the capacity to infect humans.

The reviewer has made a sharp observation from the family of sensorgram curves presented in Figure 2A and 4B, that *P. praefalciparum* RH5 appears to bind gorilla basigin better than the calculated introgressed RH5 (IntRH5) and suggests that this may have some evolutionary implications. The reviewer is correct in that the affinity for gorilla basigin is higher for *P. praefalciparum* RH5 (K_D = 2.5 μM) compared to IntRH5 (K_D = 3.0 μM) but this difference is subtle and we would be very cautious about making any further interpretation based on this small difference. One possible reason that this difference may have appeared significant is because (as is usual) we have presented the surface plasmon resonance data as a family of sensorgram curves corresponding to a dilution series of analyte concentrations, and because recombinant *P. praefalciparum* RH5 was especially well expressed, the highest concentration for *P. praefalciparum* RH5 in Figure 2A was 1200nM, twice that of the IntRH5 (600nM) used in Figure 4B. In the initial submission of the manuscript, we did not include all the different analyte concentrations for reasons of clarity but these have now all been provided in a new Table S3 that we have provided with our revised manuscript.

Reviewer #2:

In their manuscript entitled "Resurrection of the ancestral RH5 invasion ligand provides a molecular explanation for the origin of P. falciparum malaria in humans" the authors seek to show how Plasmodium parasites may have switched

> *hosts, evolving from a gorilla-specific parasite to one that can infection humans (Plasmodium falciparum). To do this, they "resurrect" the introgressed ancestral RH5 gene, which encodes the ligand which enables erythrocyte invasion by binding the basigin receptor, and conduct protein interaction assays to test binding. By doing so, they show that the ancestral P. falciparum RH5 gene can bind various receptors. Furthermore, they show that specificity to one species, or more precisely loss of specificity to a species, can arise from point mutations in the sequence. The study provides interesting molecular data to support the hypothesis that human specificity evolved via changes in rh5.*

> *The manuscript is well written and the authors provide compelling biochemical binding data. On the other hand, tests with recombinant proteins do not really tell you whether the mutations will have the same function in the context of an infection with other proteins present. Should one try swapping expression of P. falciparum rh5 with ancestral rh5 using a conditional knockdown system (for example, with a tet aptamer system, degradation domain), and test to see if this changes affinity for human erythrocytes? Such experiments are difficult and may be outside the scope of this study, however.*

We thank the referee for their positive comments on the study and their valuable suggestions for testing the function of the introgressed RH5 sequence. We did consider this experiment and consulted widely with our colleagues with experience in the genetic manipulation of the *P. falciparum* genome, and with genes encoding blood stage invasion genes in particular. The experience of trying to genetically manipulate the *rh5* locus, and particularly to make a conditional knockdown of the *rh5* gene has been a frustration for several laboratories and to our knowledge it has yet to be achieved. Conditional knockdown for other components of the RH5 complex: CyRPA and RIPR have been described (Volz *et al*. "Essential role of the PfRh5/PfRipr/CyRPA complex during *Plasmodium falciparum* invasion of erythrocytes." Cell Host and Microbe 2016 v20 p60) but for reasons that aren't fully understood, the selection of a parasite line with conditional knockdown for RH5 has not been achieved, despite the efforts of experienced laboratories.

> *In addition, the authors focus on a single 3D7 sequence and there may be other rh5 and basigin gene variants present in their respective human and parasite populations. The authors state that there are only six nonsynonymous changes between the P. falciparum rh5 and rh5 introgressed sequence and yet there seem to be more than six nonsynonymous rh5 mutations within existing P. falciparum populations (e.g. those present in PlasmoDB), and some of these variants are present in the introgressed rh5 variant (positions 148, 197, 203). Would the authors' results be different if the authors used different P. falciparum sequences? Although the 3D7 strain is the most heavily studied, it may or may*

not be a good representative for studies on species evolution. Do any of the phylogenetic trees change if one uses different strains?

To answer these questions about RH5 sequence variants (and related point from reviewer 1 above), we have now included a table based on the most recent MalariaGEN sequencing data documenting all known RH5 polymorphisms in our revised manuscript (Table S2). Based on these results, the sequence of the 3D7 strain RH5 could be considered unrepresentative of the population at position 203, which encodes a cysteine in 3D7 but most sequenced isolates (86%) encode a tyrosine (Table S2). Because this encodes a cysteine (which contains a sulfhydryl group that would normally be oxidised to form a disulfide bond) it has been investigated in some detail from the point of producing a correctly-folded recombinant protein for subunit vaccine studies. We have found that RH5 variants that contain the tyrosine at this position e.g. RH5 from the GB4 and 7G8 strains have indistinguishable binding affinity to basigin (see Supplementary Table 2 in Bustamante *et al*. "A full-length recombinant *Plasmodium falciparum* PfRH5 protein induces inhibitory antibodies that are effective across common PfRH5 genetic variants." Vaccine 2013 v31 p373) demonstrating that it plays no direct role in basigin binding. The three variants in RH5 that the reviewer specifically refers to at 148, 197 and 203 are indeed found in extant *P. falciparum* populations and we show that they have no effect on basigin binding tropisms in Figure 4B and C, as would be expected. The three other polymorphisms in the introgressed RH5 sequence at positions 200, 216 and 219 have <u>not</u> been identified in extant sequenced *P. falciparum* global isolated populations (Table S1) and we show in Figure 4B and C that it is position 200 that shows the specialisation for human basigin binding.

Minor points:

The introduction is somewhat brief and to understand this, additional background reading is needed. If length is not a constraint, this could be expanded. For example, are there additional known functions of P113, RIPR, CyRPA (only presented interactions with RH5, but what does interaction entail); knock-out studies, overexpression studies, conserved regions/homology between species?

As recommended, we have expanded the introduction to provide a more detailed background on the RH5 invasion complex and the methods used to discover them. A fully comprehensive description of the role of each component in different *Plasmodium* species would make the introduction cumbersome, and so we have focussed on the evolutionary aspects which are more pertinent to the current study. These changes are detailed in the revised manuscript file with the track changes function.

Please elaborate on how rh5 might be druggable, since this is mentioned. Would this be by disrupting protein interactions? Are there many drugs that work this way? Would an rh5 drug work against many different rh5 isolates?

RH5 is an exciting malaria blood stage vaccine target and because the interaction with the basigin receptor is essential and universally required by all tested parasite strains for erythrocyte invasion, this interaction could potentially be targeted by drugs to prevent invasion. As far as we can tell, the only mention of RH5 as a drug target within the manuscript is one of the references that we cite:
Zenonos ZA, *et al*. "Basigin is a druggable target for host-oriented antimalarial interventions". 2015 J Exp Med 212(8):1145-1151.
This study describes the isolation of a monoclonal antibody to the basigin receptor which potently prevents *P. falciparum* invasion of erythrocytes.

The focus of the manuscript is the evolutionary origin of human *P. falciparum* malaria, and because a discussion on RH5 as a drug target which is of value to others would have to be reasonably lengthy. After some debate, we have concluded that this could be seen as a distraction and so we have decided to leave this out.

Briefly state how was the sequence of the ancestral RH5 determined in the text to aid readability.

We have now modified the text in the revised manuscript in the first sentence of the results section to further explain how the ancestral sequence has been determined.

Briefly elaborate on the surface binding assay in the results. The reader currently needs to go to the methods and other publications to understand this.

We have expanded this section to provide additional information so that the reader can understand the assay without referring to other publications. It now reads:

"We confirmed the ability of the introgressed RH5 protein to specifically bind human basigin in the context of a cell membrane by using a well-characterised RH5 cell surface binding assay (20) (Fig. S3). Biotinylated RH5 was clustered around a fluorescent streptavidin conjugate to create a highly-avid fluorescent binding probe and incubated with human erythroid-like (HEL) cells. The RH5 probe bound cell surface basigin producing a shift in fluorescence when analysed by flow cytometry; the binding specificity for the basigin receptor was determined by pre-incubating the cells with an anti-basigin monoclonal antibody that prevented binding."

Briefly explain what an introgressed sequence is.

An introgression is the transfer of a portion of genome of a species into another due to an event of hybridisation between the two species followed by recurrent crossing between the hybrid (and its descendants) and the parental species. An expanded definition has now been added into the introduction of our revised manuscript which now reads:

“Introgressions are the transfer of genomic regions from one species into another due to hybridization followed by recurrent crossing between the hybrid and its descendants and the parental species. ”

The authors write, “Interestingly, the introgressed fragment encompassed two genes encoding RH5 complex components: RH5 and CyRPA, which are genetically closely linked (Fig. 1A).” but there seems to be no evidence provided in Fig 1A that RH5 and CyRPA are genetically closely linked. How many bases separate the two? Some sort of diagram or specifics would help the reader understand what is happening.

This is a good suggestion from the reviewer, and to clarify this we have prepared a new diagram which we have added to our revised manuscript as Figure S1. This new figure explains the nature of the introgressed regions and its phylogenetic characteristics.

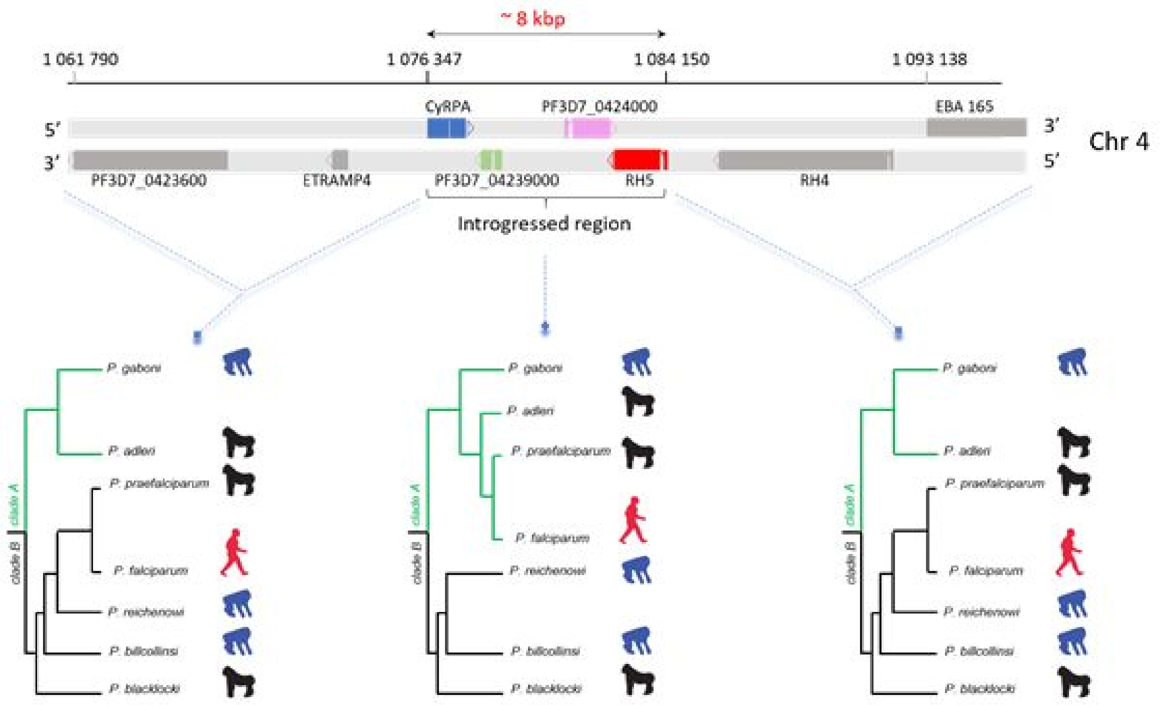

Figure S1. Schematic representation of chromosome 4 in and around the introgressed region. Each DNA strand is represented by grey bars and open reading frames encoding the named protein products are coloured. The scale indicates the equivalent position in the *P. falciparum* 3D7 reference genome. The phylogenetic topologies calculated for the introgressed and flanking sequences are provided, illustrating the extent and origin of the introgressed region.

Discussio: In the discussion of mutating residues, would be good consider that there are different variants for some of these residues in existing population genomic datasets for P. falciparum.

This is a good suggestion and we also refer to the other revisions mentioned above relating to the inclusion of Table S2. As suggested, we have modified the discussion by including additional sentences, as follows:

"While the Y197 allele is more frequent in South East Asia, the introgressed H148 allele is present in ~18% of *P. falciparum* isolates, and the Y203 allele is dominant globally, being observed in ~86% of sequenced isolates. Conversely the introgressed RH5 residues H200, R216 and Q219 have not been identified in extant *P. falciparum* populations (Table S2)."

Discussion: Mention if host tropism could be determined by proteins other than the RH and EBA family proteins?

It is indeed likely that host tropism might be determined by additional proteins, and this is particularly pertinent for the clade A parasites *P. gaboni* and *P. adleri* where we have shown it is not determined by the RH5-basigin interaction as previously assumed. Tropism could be determined by other proteins involved in the adhesion to the red blood cell like the MSP proteins but also proteins that are important at other moments of the life cycle like the invasion of the hepatocytes (e.g. Cysteine-rich protein like P36, P52...). As the reviewer suggests, we have therefore added a sentence to the discussion in our revised manuscript.

"It is, however, formally possible that other parasite proteins implicated in the adhesion to the red blood cell such as the merozoite surface proteins (MSPs) or proteins that are important at other stages of the life cycle could be involved."

Figure 1A: what do blue, black, red figures refer to (can be inferred, but better explicit)

The figures are schematics of the different hosts: gorilla, chimp and human. We have added a sentence into the figure legend of our revised manuscript to explicitly clarify this.

Figure 1A: A better description of how the dendrogram was created would be useful. Could a reference be provided?

The dendrogram was re-created from a published phylogenetic tree from the paper describing the genome sequences of all known *Laverania* species by Otto *et al*. 2018 "Genomes of all known members of the *Plasmodium* subgenus reveal paths to virulent human malaria." Nature Microbiology v3 p687, which we widely cite in the manuscript.

Figure 1B: Rh5 is drawn so the N- and C-terminal parts are separate, which is confusing. As drawn it also looks like there is no contact between the erythrocyte and the parasite, forcing one to go look at the existing literature. Perhaps a schematic diagram could be given?

The figure was purposefully drawn with the N and C-termini of RH5 separate to schematically describe more of the established molecular events since it is known that RH5 is processed into these two fragments during erythrocyte invasion. This event isn't relevant to the points discussed in our manuscript here and so to avoid any confusion, we have redrawn Figure 1B and included this modified figure in our revised manuscript (shown below).

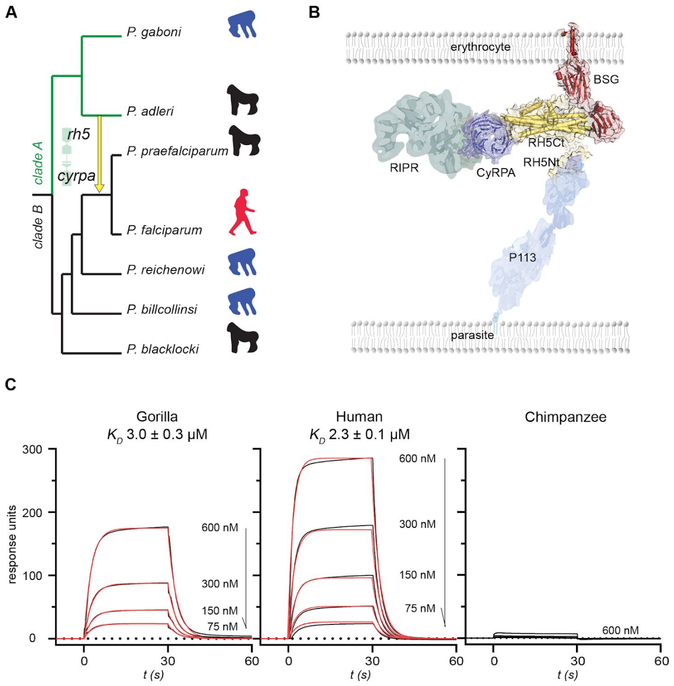

Figure 3B: Include clade information

This is a good suggestion by the reviewer and we have now added the clade information into a modified Figure 3, which we have included in our revised manuscript (shown below).

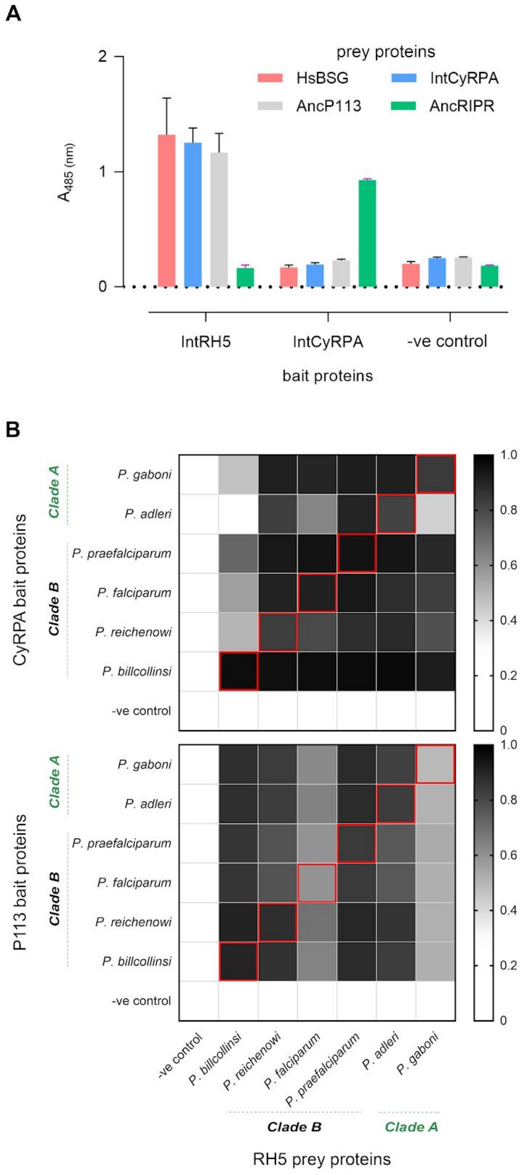

Figure 4A: where is the binding region between RH5 and basigin? Can the authors circle or highlight this region?

The original Figure 4A showed structural information on the binding region between RH5 and basigin based on the co-crystal structure with the basigin receptor shown in "ribbon" form and the electron density of RH5 rendered as a solid molecular surface. We've tried a few different ways of trying to further highlight and emphasize the contact site but after several attempts and discussion we believe that these

modifications created further confusion and so we have elected to leave the figure as it is.

Please provide Kd values plus error bars for Figure 2B.

We have now added the KD values with appropriate error handling in a modified Figure 2B which we have included in our revised manuscript (see below).

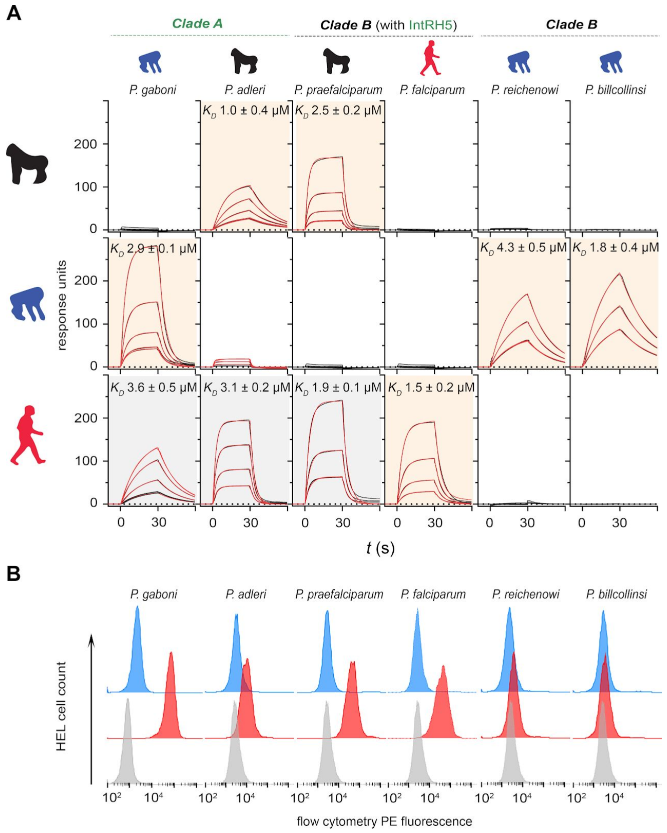

It would be useful if the authors included the Plasmodium systematic names for proteins that are discussed in Figures and text, given that there can sometimes be multiple names in the literature.

We thank the referee for their suggestions and the systematic names / accession numbers have now been added to the Materials and Methods section.

Figure S1: title is redundant with text.

Agreed - we have revised the title as indicated below. Note that this is now Figure S2 in our revised manuscript.

Figure S2. Introgressed RH5 and RH5 proteins from extant *Laverania Plasmodium* spp. The indicated *Laverania* RH5 proteins were expressed in HEK293 cells as secreted recombinant proteins with a Cd4(d3+4)-His^{6+} tag and purified by immobilised Ni^{2+} ion chromatography. Proteins were resolved by SDS-PAGE under reducing conditions and stained with Coomassie brilliant blue.

Expected molecular masses: Introgressed RH5 84.9 kDa; *P. gaboni* 85.2 kDa; *P. adleri* 84.9 kDa; *P. praefalciparum* 85.7 kDa; *P. falciparum* 84.7 kDa; *P. reichenowi* 82.5 kDa; *P. billcollinsi* 84.9 kDa.

> *Figure 2 and Table S1. It would be useful to comment that the results are from flow cytometry. The X axis could read PE fluorescence of flow sorted HEL cells as well to make the data clearer. A diagram of the experimental setup would be even better.*

Agreed. We have now modified the figure legend to include the term "flow cytometry" to make this clearer - an example is shown below.

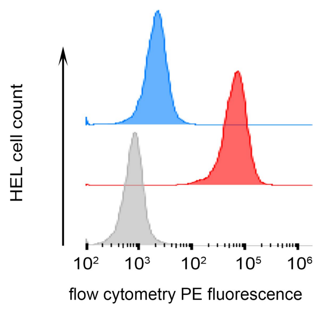

> *Reviewer #3:*

> *This very interesting article. Particularly the fact that P. gaboni shows promiscuous affinity whereas P. blacklocki that also is found in gorillas did not. Could be possible that the so-called clade B is indeed a group of parasites that can infect chimpanzees? Please clarify.*

We thank the referee for their positive comments on the manuscript. We're a little confused by the reviewer's comment that *P. blacklocki* RH5 did not exhibit promiscuous binding affinity because as we state in the methods section of the paper, we were unable to satisfy ourselves that we could express a functionally active version of *P. blacklocki* RH5 and so it was not included in our study. All attempts at expressing *P. blacklocki* RH5 led to the production of protein that was very prone to aggregation (as assessed by gel filtration) and did not exhibit any gorilla basigin receptor binding activity, nor bound *P. blacklocki* CyRPA or P113. We believe the underlying reason for this is that the published sequence for *P. blacklocki* RH5 contains errors which is perhaps unsurprising because the genome sequence from this parasite came from a single isolate which required first a selective whole genome amplification followed by a whole genome amplification (see Otto *et al*. 2018 "Genomes of all known members of the *Plasmodium* subgenus reveal paths to virulent human malaria." Nature Microbiology v3 p687). The necessary use of these genome amplification methods are likely to introduce artifactual sequencing errors which can subsequently affect protein folding and function. The question the

reviewer poses is interesting, and once more sequence data from *P. blacklocki* becomes available, we will be able to address it.

> *Nevertheless, the invasion of P. falciparum is a process that we are just starting to understand and there could be other mechanisms involved. Particularly the role of the Rh5–CyRPA–Ripr complex. I suggest checking Wang et al. 2019 Nature 565: 118–121 and discuss their results in that context. I also suggest checking Volz et al 2016 Cell Host Microbe, 20, 60–71. It will be also interesting to discuss, even as a speculation (the authors may not have a way of testing), the role that antibodies may have in inhibiting the process (Healer et al 2019 Cellular Microbiology 21: e13030) and suggest that such experiments could be of interest. Sometimes less efficient binding still suffices to mediate invasion. Although the information is valuable, it may not tell the complete story so the manuscript will improve by adding a more critical discussion of the findings.*

We thank the reviewer for these suggestions. As we show in Figure 3 of our manuscript, we thoroughly investigate the role of the Rh5-CyRPA-Ripr complex and show that the introgressed RH5 sequence is able to bind to the calculated introgressed and ancestral sequences of CyRPA, RIPR and P113 (Figure 3A). We also extend these experiments to examine the conservation of the binding of the complex components across all the extant *Laverania* parasite species (Figure 3B).

As the reviewer suggests, it would be interesting to know if antibodies may have played a role in the selection of a particular invasion pathway in the evolutionary origin of *P. falciparum* malaria. Ideally, this would involve testing a set of historical sera samples to determine if one RH5 variant may have gained the ability to avoid host immune recognition. Indeed, it is known that antibody titres to RH5 in exposed populations are low (see Douglas *et al*. "The blood-stage malaria antigen PfRH5 is susceptible to vaccine-inducible cross-strain neutralizing antibody" Nature Communications 2011 v2 p601) but do correlate with reduced susceptibility to severe malaria and parasitaemia (see: Tran *et al*. "Naturally acquired antibodies specific for *Plasmodium falciparum* reticulocyte-binding protein homologue 5 inhibit parasite growth and predict protection from malaria." J Infect Dis. 2014 v209 p789). Obtaining these historical sera samples from infected gorillas and chimps would, however, be impossible.

Finally, we agree with the reviewer that our study is unlikely to provide the complete story and our results have raised additional questions such as "what are the molecular determinants of host tropism for the clade A *Laverania* parasites *P. gaboni* and *P. adleri*?" However, we believe our study represents a very valuable contribution to our understanding of the evolutionary origin of *P. falciparum* malaria.
